# Morality, Risk-Taking and Psychopathic Tendencies: An Empirical Study

**DOI:** 10.3389/fpsyg.2022.834734

**Published:** 2022-03-03

**Authors:** Sam Cacace, Joseph Simons-Rudolph, Veljko Dubljević

**Affiliations:** ^1^Department of Public Health Sciences, University of North Carolina at Charlotte, Charlotte, NC, United States; ^2^Department of Psychology, North Carolina State University, Raleigh, NC, United States; ^3^Department of Philosophy and Religious Studies, North Carolina State University, Raleigh, NC, United States

**Keywords:** moral decision-making, moral precepts, risk-taking, psychopathy, preference for precepts implied in moral theories (PPIMT)

## Abstract

Research in empirical moral psychology has consistently found negative correlations between morality and both risk-taking, as well as psychopathic tendencies. However, prior research did not sufficiently explore intervening or moderating factors. Additionally, prior measures of moral preference (e.g., sacrificial dilemmas) have a pronounced lack of ecological validity. This study seeks to address these two gaps in the literature. First, this study used Preference for Precepts Implied in Moral Theories (PPIMT), which offers a novel, more nuanced and ecologically valid measure of moral judgment. Second, the current study examined if risk taking moderates the relationships between psychopathic tendencies and moral judgment. Results indicated that models which incorporated risk-taking as a moderator between psychopathic tendencies and moral judgment were a better fit to the data than those that incorporated psychopathic tendencies and risk-taking as exogenous variables, suggesting that the association between psychopathic tendencies and moral judgment is influenced by level of risk-taking. Therefore, future research investigating linkages between psychopathic tendencies and moral precepts may do well to incorporate risk-taking and risky behaviors to further strengthen the understanding of moral judgment in these individuals.

## Introduction

Research in empirical moral psychology has produced many findings that correlate morality, risk-taking, and psychopathic tendencies. Despite ample evidence that psychopathic tendencies and ethical decision making are negatively correlated, prior research did not sufficiently explore intervening or moderating factors. Prior literature has suggested a relationship between varying components of moral tendencies and psychopathy, but researchers have yet to discover the causal mechanisms behind these linkages (Blair, 2011). Further, prior work has also focused efforts in determining the connections between impulsivity and moral judgment, but there is paucity in the ecologically valid research in terms of the impact of risk-taking risk-taking measures on morality. The current study is the first of its kind to directly explore relationships between moral preferences, psychopathy, and risk taking.

In addition, moral psychology has suffered from a lack of standard and reliable measurement, which may contribute to the lack of evidence in supporting the connection between morality and psychopathic tendencies. Prior studies have used sacrificial moral dilemmas and Kohlbergian moral reasoning and reported diffuse and imprecise effects (Marshall et al., 2018). The current study utilizes psychometrically valid instruments in attempt to explore this research gap, and generate innovation in the understanding of the impact of risk-taking on the previously found link between psychopathic tendencies and moral preferences.

## Literature Overview

Early research into psychopathy has described the condition as a ‘moral defect’ – individuals exhibiting psychopathic tendencies were considered master deceivers, lacking moral or ethical restraints, yet behaving in public with excellent function (Cleckley, 1988). This connection between (lack of) morality and psychopathy has been repeatedly asserted during the years (see Glenn et al., 2010; Anderson and Kiehl, 2014; Glannon, 2014; Decety et al., 2015; Patil, 2015; Poppa and Bechara, 2015; Marshall et al., 2018), but due to the fact that moral intuition is a type of tacit knowledge – things people know but cannot put into words and formulate into rules all would agree on (Baron et al., 1997)– it was unclear how moral knowledge may be affected by psychopathy or how individuals with psychopathic tendencies process moral cues.

### Psychopathy and Moral Judgment

One influential study, conducted by Blair (1995), found that people admitted to psychiatric hospitals and legally categorized with Psychopathic Disorder struggle to distinguish between moral transgressions and conventional transgressions, signaling the need to further explore how people with psychopathic tendencies process *deontological* concerns (e.g., norms, rules, etc.). Blair conceptualized his findings using the developmental paradigm in research on morality (see Blair, 2011), which was largely sidelined at the turn of the century (Aharoni et al., 2012). This historical development signaled the need to develop new measures to capture the exact deficits in socio-moral judgment that people with psychopathic tendencies exhibit. Sacrificial moral dilemmas (Petrinovich and O’Neill, 1996; Christensen and Gomila, 2012) provided one way of illuminating the complexity of morality, and this line of research is still influential, despite frequent criticisms of its lack of ecological validity (Kahane and Shackel, 2010; Kahane et al., 2012; Baumard et al., 2013; Rosas and Koenigs, 2014; Schleim, 2015; Dubljević, 2017). One reason for the enduring influence of sacrificial moral dilemmas is the fact that methodological improvements such as the *perspective taking accessibility* (Martin et al., 2017, 2021) have been proposed. Either way, a study by Bartels and Pizarro (2011) has reported that participants who indicated greater endorsement of *utilitarian* solutions had higher scores on measures of psychopathy. They used sacrificial dilemmas (like the foot-bridge dilemma) presented in random order which pitted utilitarian and deontological options against each other. (The footbridge dilemma has many variations, but usually has most of the elements encapsulated by Sinnott-Armstrong, 2008).

Frank is on a footbridge over the trolley tracks. He knows trolleys and can see that the one approaching the bridge is out of control. On the track under the bridge there are five people; the banks are so steep that they will not be able to get off the track in time. Frank knows that the only way to stop an out-of-control trolley is to drop a very heavy weight into its path. But the only available, sufficiently heavy weight is a large man wearing a backpack, also watching the trolley from the footbridge. Frank can shove the man with the backpack onto the track in the path of the trolley, killing him; or he can refrain from doing this, letting the five die.

Is it morally permissible for Frank to shove the man?).

In the Bartels and Pizarro (2011) study, the subjects viewed sacrificial moral dilemmas and responded to adapted versions of personality assessments which measured markers of psychopathy. Similarly, a study by Koenigs et al. (2012) suggested that people with psychopathic tendencies are generally more willing to endorse rule violations and impersonal harms to achieve beneficial outcomes corresponding with antisocial behavior possessed by all psychopaths regardless of anxiety levels. Low-anxiety psychopaths were, however, found to be more willing to endorse personal (and more emotionally averse) harms as a means to achieving their ends – reflecting a particular deficit not shared amongst psychopathic subtypes. These studies seemed to indicate that people with psychopathic tendencies engage in utilitarian (or consequentialist) ethical decision making, while they have a harder time understanding precepts from non-consequentialist moral theories.

### Measuring Moral Preferences

Rather than relying on sacrificial moral dilemmas, a newer and more pragmatic line of research in empirical moral psychology attempts to understand the salient normative differences that laypeople have when making moral decisions by using survey methodology that is based on the operationalized principles from moral theories. This approach has precursors in the empirically-informed philosophy of Pragmatism, which posited that it is more ecologically rational to assume that, at least in lay populations, major moral theories are not viewed as incompatible rival systems, but as sources of more or less adequate precepts guiding conduct (Dewey, 1908/2009, 1966). This approach was further developed by Dubljević and Racine (2014) and empirically operationalized as the Preference for Precepts Implied in Moral Theories (PPIMT) by Dubljević et al. (2018).

The PPIMT is the first measure designed to assess respondents’ preference for the precepts implied in the three dominant moral theories (Baron et al., 1997), namely virtue ethics, deontology, and consequentialism, and it has been recently confirmed as a theoretically and psychometrically-sound model, by utilizing a combined sample of college students and Mturk respondents (Dubljević et al., 2021).

The need for such alternative approaches to the study of morality and psychopathy is readily apparent. Namely, the data from moral judgment studies on people with psychopathic tendencies that used sacrificial moral dilemmas was put into question by studies that reported the link between impulsivity and “consequentialist” responses (see e.g., Koenigs et al., 2007; Mendez, 2009; Duke and Begue, 2015). Notably, Duke and Begue (2015) reported that respondents are more likely to cause death in the footbridge dilemma and other sacrificial dilemmas when they have a higher level of alcohol inebriation. Additionally, prior work has connected impulsivity to risk-taking and risky behaviors, specifically in terms of time perspective orientation (Baumann and Odum, 2012) and behaviors likely to result in reward (Vigil-Colet, 2007), making the distinction in the literature between impulsivity as a time-oriented and situational reaction, compared to impulsivity in engaging in risky behaviors, such as gambling. Thus, a crucial question remains whether a preference for utilitarian/consequentialist ethical decision making is in fact correlated with psychopathic tendencies or if it is merely a measurement artifact of sacrificial moral dilemmas. In sum, there is a large body of research suggesting that psychopathy and moral decision making are correlated but not enough clarity if the correlations are indicative of a causal relationship or if another construct (e.g., risk-taking) is a necessary cause for the relationship between psychopathy and moral preferences.

### Relationships Between Psychopathy and Risk-Taking

One factor that may help clarify the relationship between psychopathy and moral decision making is risk-taking or risk-perception. Research suggests that while risk-taking is a broader construct applicable to many different circumstances, people with psychopathic tendencies also show a dimension of risk-taking in moral and ethical decision making. As noted above, individuals with psychopathic tendencies lean toward utilitarian approach for ethical decision making (see e.g., Koenigs et al., 2007; Mendez, 2009; Duke and Begue, 2015). The link between impulsivity and ethical decision making is viewed as a carelessness or indifference toward potential negative consequences (especially for others), often characterized as risk-taking. Boyer (2006) conceptualized risk-taking as engaging in behaviors with negative outcomes. The quintessential example of this would be Fraternity member behavior such as excessive alcohol and drug use, misogyny, and sexual assault (Seabrook et al., 2018). In this context, risk-taking is conceptualized as an action or set of actions designed to demonstrate an individual’s masculinity, performed to demonstrate prowess and social acknowledgment with little consideration of a moral or ethical decision process or the associated consequences.

Among those with psychopathic tendencies we would also expect to see higher levels of risk-taking, although we would expect that this behavior is more evaluative, resulting in a higher disregard for consequences and as a result an altered sense of ethical decision making (Hosker-Field et al., 2016). One study suggests this may represent a lower capacity for risk-perception, understanding or caring about the risks involved, which would lead to a higher level of risk-taking behavior more generally. As such, risk-taking may be a mechanism that can help clarify the relationship between psychopathy and ethical decision making.

The extant literature shows a clear paucity of explanation between psychopathic traits and moral decision-making, often either neglecting the connection between risk-taking and psychopathy established in previous literature, or lacking in appropriate measurement. To this end, the current study seeks to bridge the gap between the psychopathy and risk-taking connection, and psychopathy and moral preferences literature by examining possible relationships between psychopathy, risk-taking, and moral precepts simultaneously, using a latent modeling approach. Given prior research on the relationship between psychopathic tendencies and moral preferences, we hypothesize:

(A). Psychopathy will have a significant relationship with each subscale indicating moral precepts (Virtue Ethics, Deontology, and Consequentialism).
(B). Models which utilize Risk-Taking as a moderator between Psychopathy and Virtue Ethics, Deontology, and Consequentialism will provide a better fit to the data when compared to those that do not include moderation.

(a). Comparative models will include models where Psychopathy is the sole predictor of each PPIMT subscale; and where Psychopathy and Risk-Taking are both predictors of each PPIMT subscale with no assumed interaction effect.
(b). Comparative fit measures (AIC and LL) will be used to determine if there is a significant decrement in fit between nested and parent models.

(C). When Risk-Taking is included as a moderator between Psychopathy and Virtue Ethics, Deontology, and Consequentialism, the unmoderated relationship between Psychopathy and these moral constructs will become non-significant.
(D). The interaction between Risk-Taking and Psychopathy will be significant for all moral constructs.
(E). Simple slopes in our moderation models will indicate that the strength of the relationship between Psychopathy and moral constructs varies with levels of Risk-Taking in the current sample.

## Materials and Methods

Participants were 825 (397 female, 427 male, 1 missing gender response) college students from a large southeastern university in the United States (*M_*age*_* = 27.89; *SD*_*age*_ = 9.40). Participants agreed to participate through the informed consent process outlined by the University’s Institutional Review Board requirements. The survey was distributed *via* Qualtrics^®^, and took an average of 81.36 minutes to complete. Data were cleaned by removing linear responses, and extracting responses which failed the attention check (*n* = 15; 2.8% of total sample). For the current study, we examine responses to the Preferences for Precepts in Moral Theories ([PPIMT]; Dubljević et al., 2018), Psychopathic Personality Inventory – Revised ([PPI-R]; Lilienfeld and Widows, 2005), and Conformity to Masculine Norms ([CMNI-46]; Hammer et al., 2018) Risk-Taking subscale.

### Morality

Moral preferences were measured using the modified PPIMT (Dubljević et al., 2021), for which three subscales were derived; Virtue Ethics (4 items), Deontology (4 items), and Consequentialism (3 items). The PPIMT starts with a question “When thinking about what is moral or immoral in a situation, it is important to me whether the involved persons…” Virtue ethics items prompts the information about agents (e.g., “…have good or bad intentions”), Deontology items prompt information about the normative status of actions (e.g., “…respect or do not respect certain norms”), while Consequentialism items prompt information about outcomes (e.g., “…cause happiness or suffering”). The response scale for the PPIMT ranges from 1 = *Disagree very much* to 7 = *Agree very much*. The modified version of this measure includes a planned correlated error between items 10 and 13 within the Deontology factor, and all latent variable correlations were constrained to 0 to correspond with theory. The reliability coefficient ω for the PPIMT in the current sample was 0.92 (95% CI [0.91 | 0.93]).

### Psychopathy

Psychopathy was measured using the PPI-R (Lilienfeld and Widows, 2005), a well-validated measure consisting of 154 items (e.g., “I tell many ‘white lies”). The PPI-R is assumed to have eight subscales, for which six were used (Machiavellian Egocentricity, Rebellious Non-conformity, Blame Externalization, Social Influence, Fearlessness, and Stress Immunity). The response scale for the PPI-R ranges from 1 = *false* to 4 = *true*. The measure was scored according to the original author’s recommendations, generating t-scores for each individual subscale weighted by gender and age. These t-scores were entered as manifest variables within a single latent construct. The reliability coefficient ω within the current sample was 0.83 (95% CI [0.79 | 0.86]).

### Risk-Taking

The Risk-Taking subscale of the CMNI-46 was used to measure propensity for risky behaviors in the current study. Risk-Taking is measured using five items on a response scale from 0 = *Strongly disagree* to 3 = *Strongly agree*. These items include questions such as “I enjoy taking risks,” and “I am happiest when I’m risking danger.” The items were treated as categorical for the purposes of this study, given that scales with fewer than five anchors are best considered categorical or ordinal (Rhemtulla et al., 2012). The five-item subscale’s reliability was .83, calculated using Raykov’s (2001) estimated covariance matrix (ρ_γ_), designed for ordinal measures.

## Calculation

**FIGURE 1:**
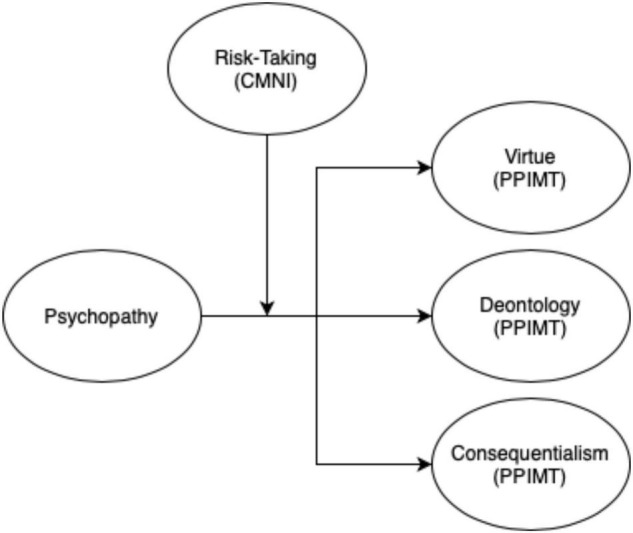
Proposed structural model of risk-taking as moderator between psychopathy and PPIMT. Only proposed structural paths are shown.

First, we ran three models to determine the predictive power of Psychopathy on the subscales of the PPIMT (Virtue Ethics, Deontology, and Consequentialism) to establish whether linear latent variable relationships exist between these constructs. The baseline models are considered exploratory to establish known patterns between Psychopathy and each moral construct from prior literature (see Figure 1). Results from these models are reported below.

To determine the validity of our stated hypotheses, we completed a structural equation model (SEM) using latent variable analysis with maximum likelihood estimation and integration algorithm with Mplus v. 8. All variables were considered latent, with Psychopathy serving as the exogenous variable, and Risk-Taking from the CMNI-46 as a moderator. Virtue Ethics, Deontology, and Consequentialism, as modified in Dubljević et al. (2021), were endogenous variables. Six total models were run since prior theoretical work on the PPIMT has designated each ethical precept as orthogonal (Dubljević et al., 2021). PPIMT items and Psychopathy items were standardized to improve interpretability of findings for each model. Each moderation model was then compared against a nested model, where both Psychopathy and Risk-Taking were regarded as exogenous variables with no moderation. All models were estimated using full information maximum likelihood, and identified using the fixed-factor variance approach, where factor variances are fixed at 1, and factor means fixed at 0.

## Results

Each of the three models where Psychopathy alone was used to predict Virtue Ethics (χ^2^(34) = 202.89, *p* < 0.0001; RMSEA = 0.078, 90%CI [0.067| 0.088], *p* < 0.05 < 0.001; CFI = 0.97; TLI = 0.95; SRMR = 0.05), Deontology (χ^2^(33) = 223.12, *p* < 0.0001; RMSEA = 0.084, 90%CI [0.073| 0.094], *p* < 0.05 < 0.001; CFI = 0.96; TLI = 0.94; SRMR = 0.05), and Consequentialism (χ^2^(26) = 157.63, *p* < 0.0001; RMSEA = 0.078, 90%CI [0.067| 0.090], *p* < 0.05 < 0.001; CFI = 0.97; TLI = 0.95; SRMR = 0.05) show acceptable fit to the data. Psychopathy has a negative relationship with Virtue Ethics (β = −0.12, *S.E.* = 0.04, *p* = 0.004), a non-significant relationship with Deontology (β = 0.08, *S.E.* = 0.04, *p* = 0.051), and a negative relationship with Consequentialism (β = −0.10, *S.E.* = 0.04, *p* = 0.02). The significant relationships found between Psychopathy and moral precepts for Virtue Ethics and Consequentialism, as defined by the PPIMT, partially supports Hypothesis A.

Compared to the parent model wherein Psychopathy and Risk-Taking are predictors with no assumed interaction (AIC = 21206.43; LL = −10551.10), the moderation model with Risk-Taking as a moderator between Psychopathy and the Virtue Ethics subscale (AIC = 21204.80; LL = −10548.40), the log likelihood ratio test indicates a significant difference between the nested moderation model and the parent model (*LRT* = 5.40; Δ*df* = 1; *p* = 0.020). Therefore, including an interaction effect does not yield a significantly poorer fit to the data when compared to the model with no interaction effect. Comparing the parent model (AIC = 21517.24; LL = −10704.62) and the nested model where Risk-Taking is a moderator between Psychopathy and Deontology (AIC = 21511.52; LL = −10700.76), the LRT indicates a significant difference between the nested moderation model and the parent model (*LRT* = 7.72, Δ*df* = 1, *p* = 0.005), with no significant decrement in fit from the nested model. Therefore, we may assume that the moderation model with Psychopathy predicting Deontology and Risk-Taking as a moderator no worse fit to the data compared to Psychopathy and Risk-Taking as non-interactive predictors. Finally, comparing the parent model (AIC = 19713.28; LL = −9806.64) where Psychopathy and Risk-Taking are treated as exogenous variables predicting Consequentialism, the moderation model (AIC = 19700.46; LL = −9799.23) indicates no reduction in fit when compared to the parent model (*LRT* = 7.41, Δ*df* = 1, *p* < 0.001). Therefore, we can assume that the model with Risk-Taking as a moderator between Psychopathy and Consequentialism fits no worse than the model where Psychopathy and Risk-Taking are exogenous predictors with no interaction. Therefore, Hypothesis B is supported for all moral constructs, such that moderation models presented no decrement in fit when compared to models without a moderation component. Loadings for Psychopathy, Risk-Taking, and each of the PPIMT subscales were significant and substantial for each model (see Tables 1, 2 for standardized loadings, standard errors, and significance values for each observed variable).

**TABLE 1:** Measurement parameters for psychopathy and risk-taking.

	Virtue ethics model		Deontology model		Consequentialism model	
	λ	*S.E.*	λ	*S.E.*	λ	*S.E.*
**Psychopathy**						
Machiavellian egocentricity	0.901	0.009	0.900	0.009	0.901	0.009
Rebellious non-conformity	0.871	0.010	0.870	0.010	0.871	0.010
Blame externalization	0.814	0.013	0.815	0.013	0.814	0.013
Social influence	0.828	0.013	0.829	0.013	0.827	0.013
Fearlessness	0.819	0.013	0.819	0.013	0.818	0.013
Stress immunity	0.615	0.023	0.618	0.023	0.615	0.023
**CMNI-46 risk-taking**						
Item 6	0.873	0.022	0.873	0.022	0.873	0.022
item 8	0.935	0.016	0.934	0.016	0.934	0.016
item 16	0.881	0.022	0.882	0.022	0.883	0.022
Item 28	0.845	0.028	0.845	0.028	0.847	0.028
Item 35	0.847	0.029	0.845	0.029	0.848	0.029

*All loadings are standardized. All loadings were significant at p < 0.001. Loadings were comparable in each model.*

**TABLE 2:** Measurement parameters for PPIMT subscales.

	λ	*S.E.*
**Virtue**		
Item 1	0.689	0.022
Item 11	0.819	0.017
Item 12	0.849	0.016
Item 15	0.709	0.021
**Deontology**		
Item 5	0.789	0.022
Item 7	0.755	0.023
Item 10	0.610	0.029
Item 13	0.679	0.026
**Consequentialism**		
Item 6	0.787	0.027
Item 4	0.704	0.028
Item 8	0.664	0.028

*All loadings are standardized and were significant at p < 0.001.*

**FIGURE 2:**
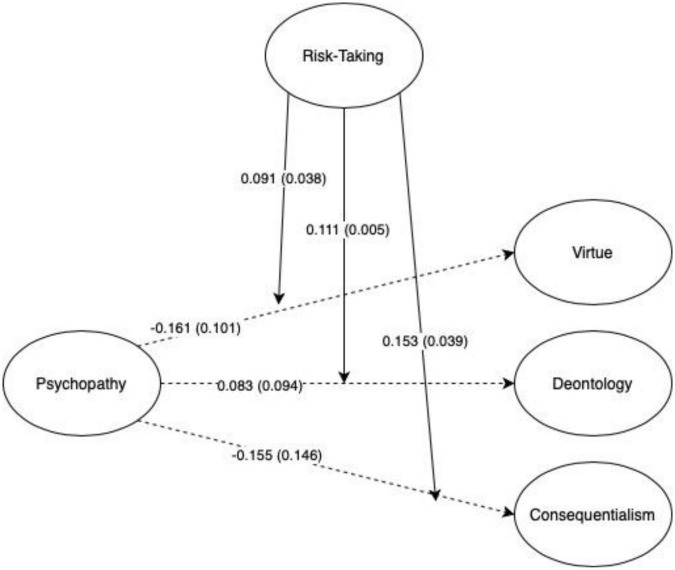
Paths between psychopathy and risk-taking interaction. Significant parameters shown with standard errors in parentheses. All paths are standardized. Significant paths shown with solid lines and non-significant paths shown with dashed line.

Neither Psychopathy nor Risk-Taking alone were significant predictors of any of the PPIMT subscales in any of the three moderation models run, indicating support for Hypothesis B. That is, when the interaction between Psychopathy and Risk-Taking was included in the model, the direct paths between Psychopathy and PPIMT subscales, and between Risk-Taking and PPIMT subscales became non-significant, thereby supporting Hypothesis C. However, all models presented significant interactions between Psychopathy and Risk-Taking on the three subscales. Specifically, the interaction between Psychopathy and Risk-Taking on Virtue Ethics, (β = 0.09, *p* = 0.018), Deontology (β = 0.08, *p* = 0.005), and Consequentialism (β = 0.15, *p* < 0.001) were all positive and significant. Additionally, Psychopathy and Risk-Taking were highly correlated in all models (*r* = 0.77, *p* < 0.001). These findings support Hypothesis D, demonstrating an interaction effect between Psychopathy and Risk-Taking for all PPIMT subscales. See Figure 2 for more details on significant paths for all moderation models.

**FIGURE 3:**
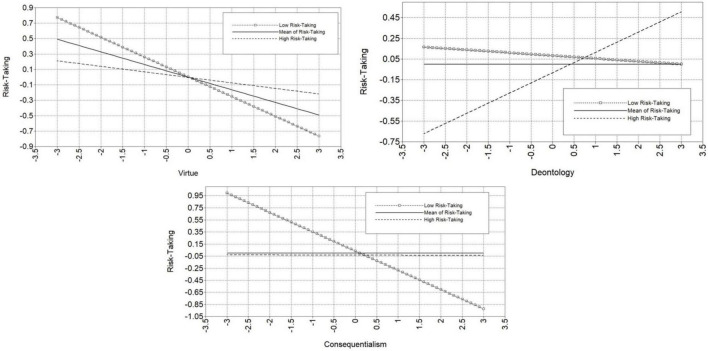
Loop plots of each moderation effect. Moderation effects shown with both moderator and outcome in standard deviations from the mean. Low risk-taking is −1*SD* from mean, and high risk-taking is + 1*SD* from mean.

Probing for interaction effects, the moderation model predicting Virtue Ethics shows that Psychopathy only predicts Virtue Ethics when Risk-Taking is at least one standard deviation below the mean (β = −0.26, *S.E.* = 0.12, *p* = 0.30). Specifically, those high in Psychopathy present with lower scores on Virtue Ethics when Risk-Taking is low. As scores on Risk-Taking increase to the mean (β = −0.16, *S.E.* = 0.10, *p* = 0.14), the relationship between Psychopathy and Virtue Ethics is reduced to non-significance. The moderation model predicting Deontology showed a significant, positive relationship between Psychopathy and Deontology when Risk-Taking was at least one standard deviation above the mean (β = 0.20, *S.E.* = 0.10, *p* = 0.04). The relationship between Psychopathy and Deontology became non-significant at lower levels of Risk-Taking. Therefore, when both Psychopathy and Risk-Taking are high, individuals tend to also score higher on Deontology. Finally, the model predicting Consequentialism showed a significant, negative relationship between Psychopathy and Consequentialism when Risk-Taking was at least one standard deviation below the mean (β = −0.32, *S.E.* = 0.13, *p* = 0.01). The relationship between Psychopathy and Consequentialism was reduced to non-significance at higher levels of Risk-Taking. Therefore, those who are high in Psychopathy but low in Risk-Taking present with higher scores on Consequentialism in the current sample. These findings support Hypothesis D; when individuals demonstrate higher Risk-Taking, then there is a significant positive relationship between Psychopathy and Deontology, such that those scoring higher in psychopathic tendencies reported greater levels of deontological moral precepts. The relationship between psychopathic tendencies and deontological moral precepts is no longer significant when participants scored at the mean or lower in Risk-Taking. In contrast, there is a significant negative relationship between Psychopathy and both Virtue Ethics and Consequentialism when Risk-Taking scores are low; the significant relationship between these constructs is eliminated for those who score at or above average in risk-taking behaviors. Thus, only individuals who score low in both risk-taking behaviors and psychopathic tendencies presented with higher scores in virtue ethics and consequentialist thinking. Significant simple slopes for each model are thus supportive of Hypothesis E. See Figure 3 for a visual representation of each moderating effect.

## General Discussion

The current study is the first of its kind to explore relationships between moral preferences, psychopathy, and risk-taking. Through these mechanisms, the results suggest that prior research on moral underpinnings has been insufficient in determining intervening factors in the relationship between psychopathic tendencies and ethical decision making. Specifically, the extant literature does not take both psychopathy and risk-taking under consideration; a significant limitation in the present literature that seeks to understand the causes of those with psychopathic tendencies to migrate toward certain components of morality. Additionally, the current study was the first of its kind to investigate these relationships using a latent variable modeling approach, which considers Psychopathy, Risk-Taking, and moral constructs to be unobserved variables with error. The latent variable modeling approach permits more realistic, generalizable interpretations over prior work in the field given the lack of direct observation in all investigative constructs.

Specifically, the current study found support for Risk-Taking serving as a moderator between Psychopathy and Virtue Ethics, Deontology, and Consequentialism. The relationship between Psychopathy and both Virtue Ethics and Consequentialism is only significant in the current sample when participants reported fewer risk-taking behaviors, indicating that either those who present with more psychopathic tendencies, but show restricted risk-taking, are less likely to hold consequentialist or virtue ethics moral precepts. In contrast, Deontology’s positive relationship with Psychopathy only exists when participants report higher-than-average risk-taking behaviors. Thus, those who have greater penchant for psychopathic tendencies are more deontological in their thinking only when they engage in more risk-taking behaviors. This finding supports prior work by Duke and Begue (2015), which cautions against relying on simple and indirect measures of morality. It also validates early findings by Blair (1995), by providing a more nuanced interpretation of the effect of psychopathic tendencies on rule breaking. Finally, our study illuminates a glaring problem reported in a recent meta-analysis of studies on psychopathy and moral judgment (Marshall et al., 2018). Namely, Marshall et al. (2018) located published and unpublished works that examined the relation between psychopathy and the three examination methods: sacrificial moral dilemmas, Kohlbergian moral reasoning, and Moral Foundations questionnaire. Looking at the relationship between Sacrificial Moral Dilemmas and Kohlbergian Moral reasoning, these showed minor discrepancies representing the fact that moral reasoning tasks showed little variance compared to that of decision-making tasks of normal controls. Furthermore, with the Moral Foundations Questionnaire the authors noted a slightly stronger, but not significant, magnitude in the Harm subscale for psychopathic individuals showing less concern about harm compared to other foundations. In conclusion, their study represented two meta-analyses, with the first suggesting a weak relationship between psychopathy and commonly used measures of moral judgment. The second suggested that psychopathic individuals have different moral preferences than those who are not psychopathic. Marshall and colleagues strongly encouraged further research examining the relationship between psychopathy, especially at the sub-dimension level, and moral judgment, while acknowledging the weak ecological validity of moral judgment indices. They specifically note “researchers should examine moral judgment using alternative measures of moral decision-making that better detects differences in moral judgment and are more externally valid” (Marshall et al., 2018, p.48). However, it should be noted that while the current study supports prior work on impulsivity and risky behaviors serving as a moderating influence on the links between psychopathic tendencies and moral precepts, prior work has identified the importance of evaluating situational factors when considering risky behaviors and impulsivity (e.g., Kusev et al., 2020; Teal et al., 2021). Lending further evidence toward our supported hypothesis, psychopathic tendencies may contribute to moral decision-making only when combined with higher levels of clinically risky behaviors, such as problem gambling (Teal et al., 2021). Future scholars may consider separating types of risky behaviors according to severity and type to identify specific moderating effects between psychopathic tendencies and moral precepts.

Our study offers evidence that PPIMT, a new, more nuanced and ecologically valid measure of moral judgment could better explain the specific deficits in socio-moral judgment of neurodiverse populations, especially when paired with valid measures of behavioral impulsivity and risk-taking. We encourage further research using the PPIMT measure, conducted by unrelated researchers, and with other populations exhibiting deficits in socio-moral judgment and behavior.

## Limitations

While our study provides new insights into the potential moderating relationship of risk-taking between psychopathy and moral precepts, there are some limitations to note in the current study. Social scientists in a variety of contexts have noted that cross-sectional measurement suffers from indetermination of stability across time and situations (e.g., Bleidorn et al., 2021), and instead recommend tempering expectations to restrict interpretations to immediate interactions and outcomes (Kelley and Turner, 2014). Thus, the present study can only conclude risk-taking’s moderating effect at the time of measurement for the sample. Additionally, prior work has found that risky behaviors and impulsivity are sensitive to context (e.g., Kusev et al., 2020; Teal et al., 2021). Additionally, impulsivity tends to inform risky behavior when dysfunctional impulsivity (Vigil-Colet, 2007) or potential clinical levels of problem behaviors exist within the individual (Baumann and Odum, 2012; Kusev et al., 2020). Therefore, future work on psychopathic tendency’s relationship to moral precepts may consider specific contexts for impulsive decision-making and severity of risk-taking behavior within-person.

## Data Availability Statement

The raw data supporting the conclusions of this article will be made available upon written request by the authors, without undue reservation.

## Ethics Statement

The studies involving human participants were reviewed and approved by North Carolina State University IRB. The patients/participants provided their written informed consent to participate in this study.

## Author Contributions

SC contributed to the data curation, formal analysis, wrote the original draft, and review and editing. JS-R contributed to the data curation, and wrote the review and editing. VD contributed to the conceptualization, data curation, formal analysis, funding acquisition, investigation, methodology, project administration, resources, supervision, validation, visualization, wrote the original draft, and review and editing. All authors contributed to the article and approved the submitted version.

## Conflict of Interest

The authors declare that the research was conducted in the absence of any commercial or financial relationships that could be construed as a potential conflict of interest.

## Publisher’s Note

All claims expressed in this article are solely those of the authors and do not necessarily represent those of their affiliated organizations, or those of the publisher, the editors and the reviewers. Any product that may be evaluated in this article, or claim that may be made by its manufacturer, is not guaranteed or endorsed by the publisher.
